# Unraveling a Rare Case of Epidural Extramedullary Hematopoiesis in a Patient With Transfusion-Dependent Beta Thalassemia Presenting With Spinal Cord Compression

**DOI:** 10.7759/cureus.56352

**Published:** 2024-03-17

**Authors:** Raed Masalma, Thabet Zidan, Khalil M Abualhumos, Dalia Hamayel, Ziad Abukhalil, Ahmed T Ghanem, Adnan Mousa, Maroun Helou, Wesam Tamimi, Mahdi Al-Sayed Ahmad

**Affiliations:** 1 Faculty of Medicine and Health Sciences, An-Najah National University, Nablus, PSE; 2 Department of Neurosurgery, Palestine Medical Complex, Ramallah, PSE

**Keywords:** radiotherapy, decompressive surgery, spinal cord compression, thalassemia, extramedullary hematopoiesis

## Abstract

Thalassemia is known to induce extramedullary hematopoiesis (EMH), which is a compensatory mechanism in which the body forms blood cells outside the bone marrow. While EMH typically affects organs such as the spleen and liver, there are rare instances where it leads to spinal cord compression (SCC) in the epidural space.

A 31-year-old male patient with transfusion-dependent beta thalassemia presented with numbness and bilateral limb weakness due to EMH. Neurological examination revealed increased tone in both legs, reduced power, loss of crude touch and pain sensation, and increased deep tendon reflexes. Magnetic resonance imaging (MRI) indicated a lobulated soft tissue structure in the posterior dural intrathecal space causing SCC. Laminectomy of the T2-T8 vertebrae was done, after which the lesion was identified and completely removed. Post-surgery, significant neurological improvements were observed in both motor and sensory functions. Thalassemia patients presenting with symptoms of SCC should be investigated for the presence of epidural EMH. Treatment options include decompressive surgery, blood transfusions, hydroxyurea, and radiotherapy.

## Introduction

Thalassemia is an autosomal recessive inherited hematological disorder characterized by chronic anemia [1]. It is prevalent in regions like the Middle East, the Mediterranean region, and North Africa [1]. Chronic hemolytic anemia often triggers compensatory reactions, most notably, extramedullary hematopoiesis (EMH) [2]. This phenomenon involves the generation of hematopoietic elements outside the bone marrow and peripheral blood, typically occurring in response to different hemolytic disorders such as thalassemia, polycythemia vera, myelofibrosis, leukemia, lymphoma, and following bone marrow irradiation [2]. While EMH commonly affects the spleen, liver, and lymph nodes, the occurrence of spinal cord compression (SCC) due to EMH in the epidural space is a rare condition [2]. The management of SCC due to EMH remains a matter of debate, encompassing different interventions such as decompressive surgery, blood transfusion, and radiotherapy [2]. 

In this report, we present a case of a 31-year-old male patient with transfusion-dependent beta thalassemia who presented with symptoms of SCC due to EMH. The condition was successfully treated with surgical decompression.

## Case presentation

A 31-year-old male patient, who is a known case of transfusion-dependent beta thalassemia, was referred to our hospital complaining of interscapular pain and bilateral lower limb weakness. The pain started five months ago, initially relieved by non-steroid anti-inflammatory drugs (NSAIDs) and muscle relaxants. However, over the last month, it worsened and became associated with progressive numbness and weakness in both lower limbs. Neurological examination showed difficulty walking without assistance, increased tone in both lower limbs, and decreased power bilaterally, particularly in hip flexion graded at 2+/5. Deep tendon reflexes were rated at +3 bilaterally in the lower limbs. Sensory examination indicated a loss of crude touch and pain sensation, but intact proprioception in both lower limbs. Patients maintained continence for urine and stool, and there were no muscle wasting or fasciculations. Upper limbs examination showed no abnormalities. The patient is splenectomized and his hemoglobin (HGB) level on admission was 11.3 g/dL, mean cell volume (MCV) 78.5 fL, mean cell hemoglobin (MCH) 29.7 pg, mean cell hemoglobin concentration (MCHC) 37.8 g/dL, and red blood cells (RBC) 3.81 M/uL. 

**Figure 1:**
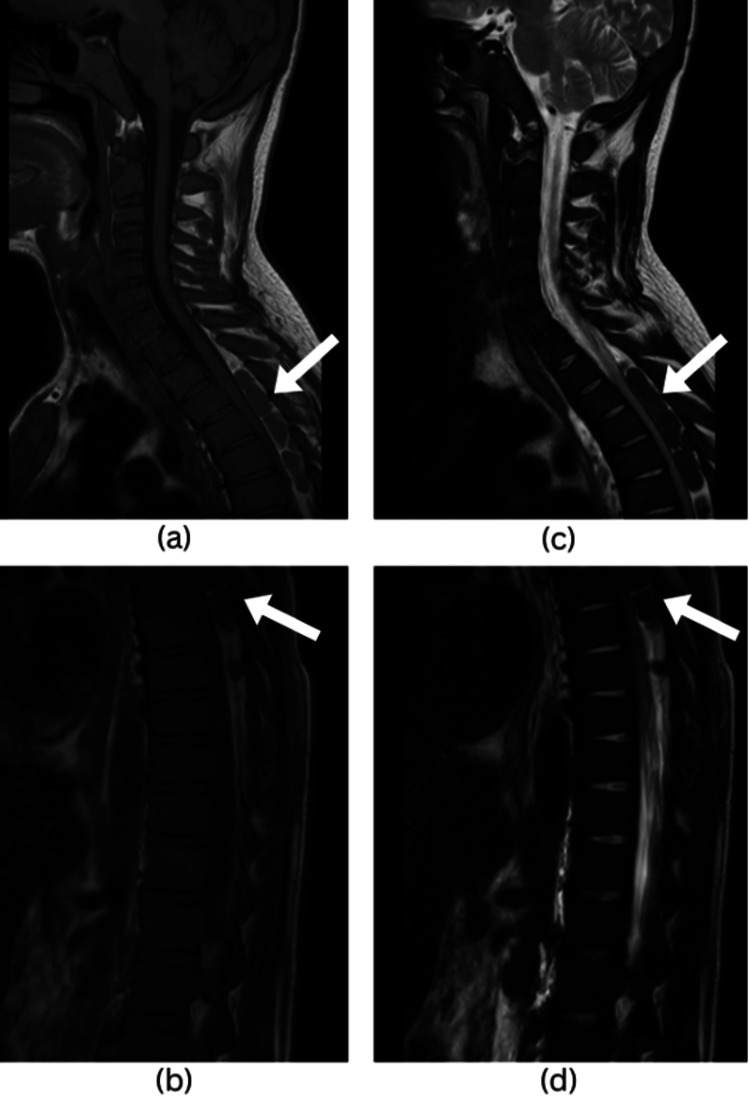
Pre-op MRI of the dorsal spine. Sagittal T1-weighted images (a,b) and sagittal T2-weighted images (c,d) show an epidural isointense lesion (marked by arrows), located posteriorly and compressing the spinal cord from T3 to T8.

The magnetic resonance imaging (MRI) showed a lobulated soft tissue structure in the posterior dural intrathecal space extending from T3 to T8 levels and compressing the cord (Figure 1). The structure is isointense on both pulse sequences (T1W/T2W) and showed mild homogeneous enhancement after intravenous (IV) contrast administration, indicating possible EMH.

A decision to proceed with surgery was made to excise the lesion, and the patient was transferred to the operating room. The patient was put in a prone position, a skin incision was made from T2 down to T8, and dissection of the paraspinal muscles on both sides was performed. The spinous processes from T3 to T8 were removed, and bilateral T3-T8 laminectomy was carried out. The bone exhibited hypertrophy and hyperplasia, and it showed excessive bleeding. Following the removal of the ligamentous complex, the lesion compressing the spinal cord posteriorly from T3-T8 was identified. Dissection using a blunt dissector upwards from T8 was conducted, resulting in the complete resection of the lesion. Specimens of both bone and soft tissue were sent for pathological examination.

**Figure 2:**
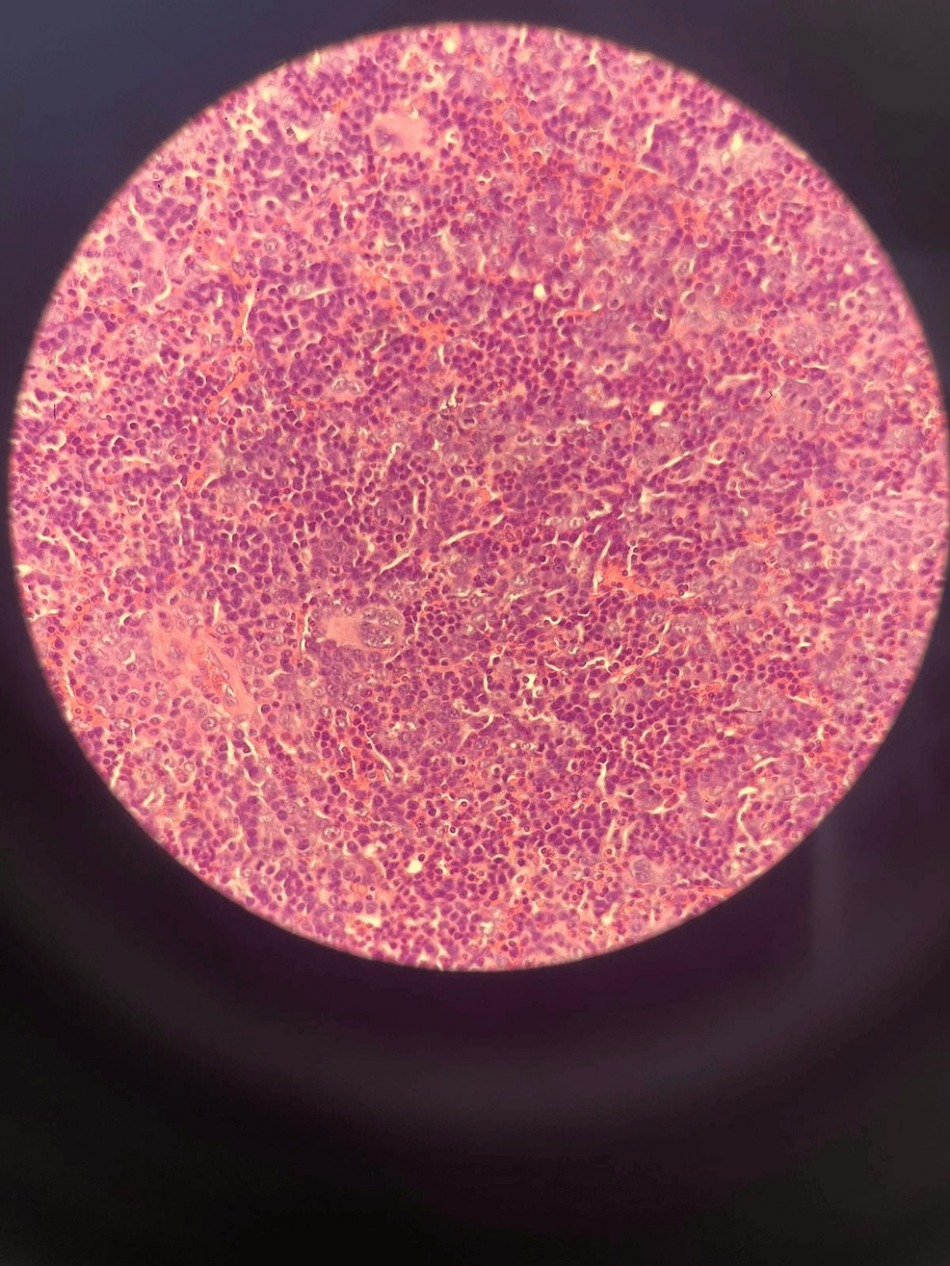
Microscopic examination of the pathology specimen.

Pathological examination showed tissue comprising all hematopoietic elements, i.e. myeloid, erythroid and megakaryocytic (Figure 2). These features are most consistent with epidural EMH with prominent erythroid precursors. No evidence of lymphoma or epithelial malignancy was detected.

Neurological examination after surgery demonstrated significant improvement. Motor examination indicated that lower limbs power improved to grade 4/5, sensory examination demonstrated improvement in crude touch and pain sensation compared to pre-operation. 

**Figure 3:**
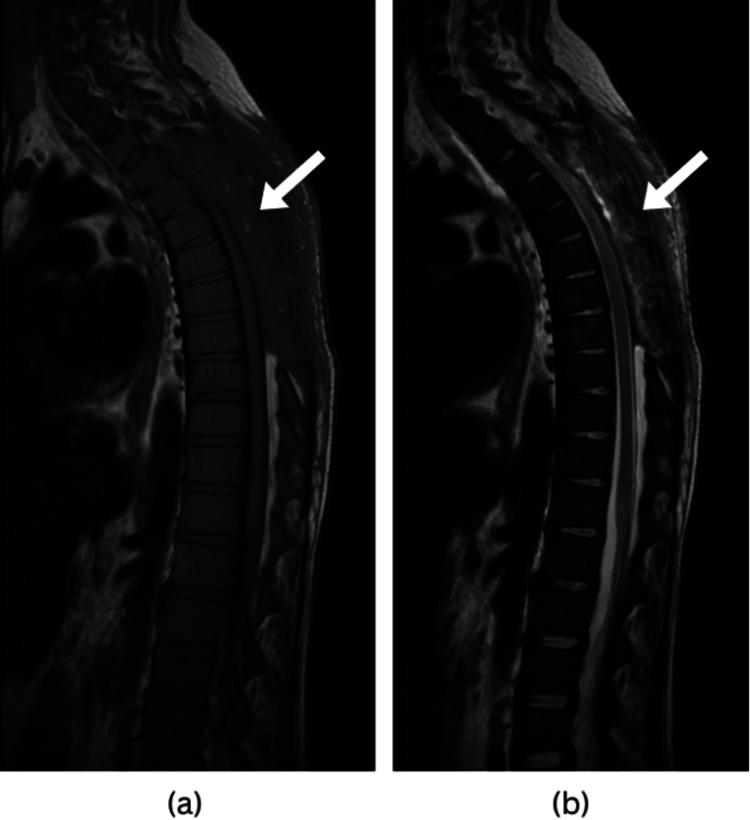
Post-op MRI of the dorsal spine. Sagittal T1-weighted image (a) and sagittal T2-weighted image (b), a laminectomy is evident, with the spinal processes absent from T3 to T8 (indicated by arrows), along with the removal of the previously observed epidural lesion. There is no evidence of spinal cord compression.

Follow-up after two months showed improvement in the patient's clinical condition, neurological exam showed no deficits and no signs of spinal cord compression. An MRI was ordered and showed a laminectomy with the absence of the spinal processes from T3 to T8 (Figure 3). It also showed the absence of the epidural lesion and the lack of cord compression. The patient was referred to the hematology team for further follow-up and management.

## Discussion

**Figure 4:**
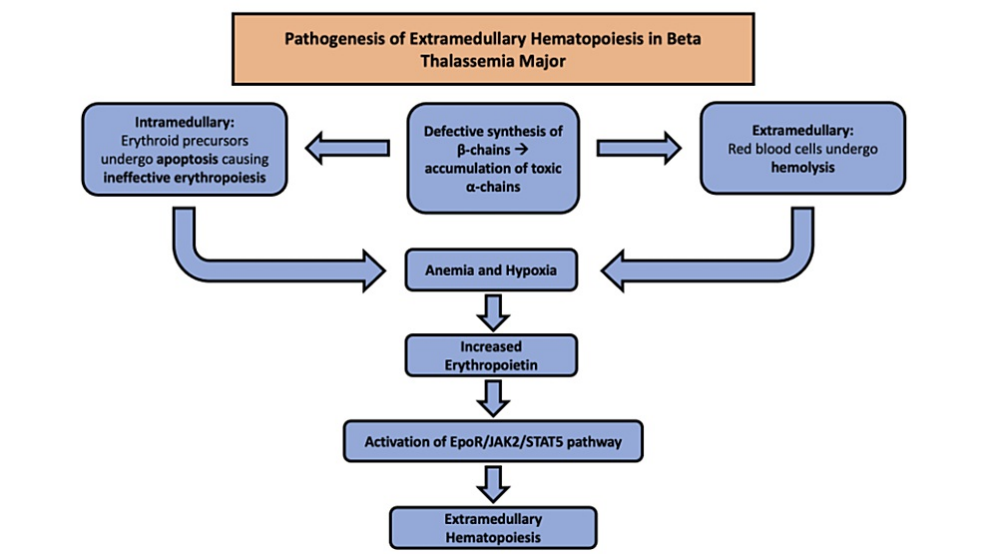
Pathogenesis of EMH in beta-thalassemia major patients. EpoR: erythropoietin receptor, JAK2: Janus kinase 2, STAT5: signal transducer and activator of transcription 5, EMH: extramedullary hematopoiesis

EMH results from defective erythropoiesis, leading to compensatory growth of hematopoietic tissue outside the bone marrow. (Figure 4) [3]. This phenomenon has been documented in chronic hemolytic anemias, myeloproliferative disorders, myelophthisic diseases, hepatocellular cancer, and disorders causing bone marrow disruption [4,5]. Its highest prevalence is observed in beta-thalassemia patients, notably thalassemia intermedia at 20%, with a male-to-female predominance of 5:1 and a usual onset during the fourth decade of life. It rarely occurs in transfusion-dependent thalassemia major, with incidence rates at 1% [4]. This discrepancy in prevalence between transfusion-dependent and non-transfusion-dependent thalassemia is probably attributed to the transfusions suppressing the need of EMH to compensate for anemia [2]. 

EMH typically involves organs responsible for hematopoiesis during fetal life, mainly the spleen and liver [2]. Reports of EMH occurrence in the epidural space are infrequent; however, there is a tendency for the thoracic spine to be involved, as in our case [6]. The cause for this is not yet clear, though it is hypothesized that it arises from hematopoietic precursor cell rests present in the spinal canal, notably in the posterior thoracic epidural space [7]. Other suggested theories include the possibility of hematopoietic tissue embolism into the intraspinal space, as well as direct extension of hematopoietic tissue from the vertebral body medulla [7]. The first two proposed theories of embryonic rests and embolism align more with our case since MRI did not show hematopoietic tissue extending or within the vertebral bodies of the same density and intensity as the epidural hematopoietic tissue. 

Paraspinal EMH can be incidentally found and is asymptomatic in more than 80% of cases [8]. However, when symptomatic, it can be debilitating due to compressive effects on the neural tissue. Patients experiencing spinal cord compression can present with a multitude of symptoms such as back pain, paraplegia, paraparesis, impaired sensation and reflexes, spasticity, and incontinence [8]. The major signs and symptoms found in our patient were consistent with an upper motor neuron lesion and sensory loss of only crude touch and pain sensation, without incontinence. It is noteworthy that patients with thoracic epidural EMH are more likely to show signs and symptoms due to the smaller diameter of the spinal canal at this level relative to other areas [9]. 

Diagnosis should be made promptly, as delays in treatment can cause permanent neurological damage [10,11]. Thus, age, medical history, and examination are crucial to rule out other causes of spinal cord compression which may also necessitate a biopsy, and confirm EMH as an anticipated consequence of the patient’s condition [10,11]. In the past, diagnosis was suspected when well-demarcated masses were observed on plain radiographs, in the presence of specific osseous changes resulting from chronic anemic state [12,13]. Nowadays, magnetic resonance imaging MRI is the preferred diagnostic modality, replacing the need for both CT and myelography due to associated radiation risk and invasiveness, respectively [2]. MRI is also superior as it can precisely indicate the extent of involvement in surrounding tissues [7]. Needless to say, biopsy is the gold standard for diagnosis, but it comes with the risk of a devastating hemorrhage and must be avoided unless the clinical picture and radiological imaging are equivocal; therefore it was not performed for our case [14].

Regarding management, clear guidelines have not been established yet due to the rarity of this condition [15]. Treatment may involve the solitary use or a combination of various modalities which include blood transfusions, hydroxyurea, radiotherapy, and surgery. Determining the choice of treatment is dependent on a multidisciplinary team following the case, which decides based on severity and acuity of symptoms, patient’s clinical condition, and recurrence after previous treatments.

Mild cases of EMH and cases such as pregnancy, where surgery and radiotherapy are contraindicated, could be managed with blood transfusions. This approach also serves as a diagnostic modality since the response to treatment justifies the etiology. However, the limitation of this option in treating moderate to severe cases arises from the recurrence of symptoms and the inability to reverse pre-existing compression [16]. Blood transfusion is usually required in high amounts and is often referred to in literature as “hyper-transfusion”, this results in the increased risk of various transfusion-related issues, including but not limited to circulatory overload, iron overload, infections, hemolytic or non-hemolytic reactions, and alloimmunization [2,16,17]. 

Hydroxyurea has myelosuppressive properties and has been documented for use alone in patients with alloimmunization, preventing them from receiving transfusions. It is also utilized as an adjunct or in combination with radiotherapy and transfusions [2,18].

Neurological deficits, acute and severe cases, along with recurrence have been indications seen in literature that necessitate surgical decompression [19]. Surgery implicates an invasive and possibly decisive treatment measure, aiding in the histopathologic diagnosis of the tissue. However, it may come at the expense of incomplete mass resection and recurrence, spinal instability especially if laminectomy is performed over a lengthy segment, excessive bleeding, and infections. Furthermore, it carries the risks associated with general anesthesia.

Radiotherapy is becoming an increasingly favored choice of treatment for radiosensitive tissue, for it is less invasive [7]. One study showed that the size of EMH decreases by 16.4% immediately after radiotherapy [20]. The downsides of it remain the need for establishing a definite diagnosis, the possibility of anemia exacerbation following decreased hematopoiesis in adjacent tissue, alongside recurrency amenable to multiple dosing [7,21]. In addition, radiotherapy requires the concomitant use of steroids to prevent edema that may occur with initial use, as edema intensifies compressive effects, leading to further neurological deterioration [2].

In the case described in this report, the decision to proceed with surgery was based on the assessment of the neurosurgery team that radiation would take time to decompress the cord. This delay was deemed unacceptable due to the presence of truly rapidly progressive neurological deficits necessitating swift intervention to prevent potential ischemic changes. The surgery was successful, as the subsequent examination indicated improved outcomes in all aspects compared to the patient’s condition before the operation.

## Conclusions

Patients with thalassemia who present with symptoms of SCC should undergo investigation for epidural EMH. MRI serves as the diagnostic imaging modality of choice, with biopsy being the gold standard. Treatment options encompass decompressive surgery, blood transfusion, radiotherapy, and hydroxyurea. The seriousness of this situation underscores the importance of prompt diagnosis, as delays may result in permanent neurological damage. 
